# Supplementary material for: Modulation of AggR levels reveals features of virulence regulation in enteroaggregative E. coli
Source: Commun Biol. 2021 Nov 16;4:1295. doi: 10.1038/s42003-021-02820-9 (PMC8595720; doi:10.1038/s42003-021-02820-9)
Supplement: Supplementary file 2 — Supplementary information [file 42003_2021_2820_MOESM2_ESM.pdf]

Modulation of AggR levels reveals features of virulence regulation in enteroaggregative *E. coli*

Alejandro Prieto[1], Manuel Bernabeu[1], José F. Sánchez Herrero[2], Anna Pérez-Bosque[3,4], Lluïsa Miró[3,4], Christine Bäuerl[5], Carmen Collado[5], Mário Hüttener[1*] and Antonio Juárez[1,6*]

[1]Department of Genetics, Microbiology and Statistics, Universitat de Barcelona, Barcelona, Spain.

[2]High Throughput Genomics and Bioinformatics Facility, Institut Germans Trias i Pujol, Badalona, Spain

[3]Department of Biochemistry and Physiology, Universitat de Barcelona, Barcelona, Spain.

[4]Institut de Nutrició i Seguretat Alimentària, University of Barcelona, Barcelona, Spain.
[5]Institute of Agrochemistry and Food Technology, National Research Council (IATA-CSIC), Paterna, Valencia, Spain

[6]Institute for Bioengineering of Catalonia, The Barcelona Institute of Science and Technology, Barcelona, Spain.

*Corresponding authors: Prof. Antonio Juárez (ajuarez@ub.edu) and Dr. Mário Hüttener (mhuttener@me.com).

Supplementary Table 1. List of genes belonging to the *aggR* regulon identified in previous studies[1,2]. The positive fold change values shown were obtained for strain 042 *aggR+FRT3'UTR* compared to wt.

***aggR* regulon (according to[1,2])**	
**NCBI identifier locus tag**	**Fold change**
EC042_pAA105 (pseudo gene)	6,3
EC042_RS26280/EC042_pAA010 (dispersin export ABC transporter ATP-binding protein AatC)	3'6
EC042_RS26265/EC042_pAA007 (dispersin export ABC transporter permease subunit AatP)	3,2
EC042_RS26275/EC042_pAA009 (dispersin export-associated protein AatB)	3,2
EC042_RS11835/EC042_2219 (inverse autotransporter)	3,2
EC042_RS26510/EC042_pAA060 (AggR-activated transcriptional regulator Aar)	2,9
EC042_RS26270/EC042_pAA008 (dispersin export ABC transporter outer membrane protein aatA)	2,8
EC042_RS05935/EC042_1127 (biofilm formation regulator BssS)	2,8
EC042_RS23675/EC042_4430 (c-di-GMP phosphodiesterase PdeC)	2,8
EC042_RS27190/EC042_pAA022 (putative hexosyltransferase CapU)	2,8
EC042_RS26460/EC042_pAA046 (aggregative adherence fimbriae II chaperone AafD)	2,4
EC042_RS26345/EC042_pAA023 (virulence factor VirK)	2,3
EC042_RS26465/EC042_pAA048 (aggregative adherence fimbriae II major subunit aafA)	2,3
EC042_RS26240/EC042_pAA003 (class 1 isoprenoid biosynthesis enzyme)	2,3
EC042_RS26245/EC042_pAA004 (isopentenyl-diphosphate delta-isomerase)	2,3
EC042_RS26325 (hypothetical pseudoprotein)	2,1
EC042_RS30530/EC042_pAA055 (dispersinaap)	2,0
EC042_RS26515 (IS630 family transposase)	2,0
EC042_pAA047 (hypothetical protein)	2,0
EC042_RS26335/EC042_pAA021 (polysaccharide deacetylase family protein)	2,0
EC042_RS26330/EC042_4581 (hypothetical protein)	1,8
EC042_RS17030/EC042_3187 (AAA family ATPase)	1,8
EC042_4581 (hypothetical protein)	1,8
EC042_RS24360/EC042_4562 (type VI secretion system contractile sheath small subunit tssB)	1,8
EC042_RS24365/EC042_4563 (type VI secretion system contractile sheath large subunit tssC)	1,8
EC042_RS24370/EC042_4564 (type VI secretion system protein AaiC/Hcp2)	1,7

EC042_RS24375/EC042_4565 (putative type VI secretion protein)	1,7
EC042_RS16990 (hypothetical protein)	1,6
EC042_RS26470/EC042_pAA050 (IS1-like element IS1A family transposase)	1,6
EC042_RS30500/EC042_pAA006 (pseudogene - transposase)	1,6
EC042_RS30525/EC042_pAA053 (IS3 family transposase)	1,6
EC042_RS26480/EC042_pAA052 (aggregative adherence transcriptional regulator AggR)	1,6
EC042_RS30410/EC042_4581A (transposase)	1,5
EC042_RS17010/EC042_3184 (hypothetical protein)	1,5
EC042_RS24435/EC042_4577 (type VI secretion system ATPase TssH)	1,5
EC042_RS16995/EC042_3181 (PerC family transcriptional regulator)	1,5
EC042_RS24385/EC042_4568 (type VI secretion system baseplate subunit TssG)	1,5
EC042_RS24390/EC042_4569 (type VI secretion system tip protein VgrGTssI)	1,5
EC042_RS17015 (pseudogene)	1,5
EC042_RS12055/EC042_2249 (type IV toxin-antitoxin system toxin CbtA)	1,5
EC042_RS24430/EC042_4576 (putative type VI secretion protein)	1,5
EC042_4581B (hypothetical protein)	1,4
EC042_pAA033 (transposase)	1,4
EC042_RS24490/EC042_4587 (IS110-like element ISEc45 family transposase)	1,4
EC042_RS24460/EC042_4582 (hypothetical protein)	1,4
EC042_RS24380/EC042_4566 (type VI secretion system baseplate subunit TssF)	1,4
EC042_RS17005/EC042_3183 (phosphoadenosine phosphosulfate reductase)	1,4
EC042_RS26390/EC042_pAA031 (aggregative adherence fimbria II usher protein aafC)	1,4
EC042_RS28315/EC042_3188 (transposase)	1,3
EC042_pAA005A (conserved hypothetical protein)	1,3
EC042_RS24475/EC042_4584 (IS110 family transposase)	1,3
EC042_RS24415/EC042_4574 (type VI secretion system baseplate subunit TssK)	1,3
EC042_RS24400/EC042_4571 (type VI secretion protein)	1,3
EC042_RS24395/EC042_4570 (putative type VI secretion protein)	1,3
EC042_RS24405/EC042_4572 (type VI secretion system protein TssA)	1,3

Supplementary Table 2. List of the 45 genes exhibiting the highest fold change values in strain 042 *aggR+FRT3'UTR* compared to their expression in the wt 042 strain.

E. coli 042 *aggR+FRT3'UTR* vs *E. coli* 042 WT	
**NCBI identifier locus tag**	**Foldchange**
EC042_RS23795 (hypothetical protein)	11,3
EC042_RS16305 (hypothetical protein)	9,4
EC042_RS17065 (CPBP family intramembrane metalloprotease)	9,4
EC042_RS21655 (hypothetical protein)	7,5
EC042_RS16275 (hypothetical protein)	6,6
EC042_RS16360 (invasion protein)	5,7
EC042_RS16280 (hypothetical protein)	5,7
EC042_RS09160 (cell division inhibition protein DicB)	5,0
EC042_RS12795 (hypothetical protein)	5,0
EC042_RS05725 (trifunctional transcriptional regulator/proline dehydrogenase/L-glutamate gamma-semialdehydede hydrogenase)	4,9
EC042_RS09020 (hypothetical protein)	4,7
EC042_RS29440 (hypothetical protein)	4,7
EC042_RS12450 (PTS galactitol transporter subunit IIB)	4,5
EC042_RS07860 (phage lysis protein EssD)	4,4
EC042_RS10140 (N-succinylarginine dihydrolase AstB)	4,3
EC042_RS10145 (succinylglutamate-semialdehydedehydrogenase AstD)	4,3
EC042_RS10135 (succinylglutamate desuccinylase AstE)	4,2
EC042_RS29405 (hypothetical protein)	4,1
EC042_RS22490 (fatty acid oxidation complex subunit alpha FadB)	4,1
EC042_RS02130 (hydrogen peroxide resistance inhibitor IprA)	4,1
EC042_RS22485 (acetyl-CoA C-acyltransferase FadA)	3,9
EC042_RS05770 (phosphate starvation-inducible protein PhoH)	3,9
EC042_RS03040 (hypothetical protein)	3,8
EC042_RS15365 (glutaredoxin-like protein NrdH)	3,8
EC042_RS09860 (MFS transporter)	3,8
EC042_RS09040 (YdfR family protein)	3,8
EC042_RS03755 (K(+)-transporting ATPase subunit F)	3,8
EC042_RS05765 (hypothetical protein)	3,7
EC042_RS19645 (type 1 fimbrial protein)	3,6
EC042_RS26280 (dispersin export ABC transporter ATP-binding protein AatC)	3,6
EC042_RS03060 (hypothetical protein)	3,6
EC042_RS10155 (succinylornithine/acetylornithine transaminase AstC)	3,6
EC042_RS10150 (arginine N-succinyltransferase AstA)	3,5
EC042_RS20725 (hypothetical protein)	3,5
EC042_RS16260 (helix-turn-helix transcriptional regulator)	3,5
EC042_RS01925 (hypothetical protein)	3,5
EC042_RS17395 (malate synthase G GlcB)	3,5
EC042_RS09850 (YdiL family protein)	3,3
EC042_RS29570 (hypothetical protein)	3,3
EC042_RS23860 (phosphonate C-P lyase system protein PhnG)	3,3
EC042_RS21385 (hypothetical protein)	3,2
EC042_RS26265 (dispersin export ABC transporter permease subunit AatP)	3,2
EC042_RS26275 (dispersin export-associated protein AatB)	3,2
EC042_RS15285 (carbon starvation induced protein CsiD)	3,2

EC042_RS15295 (NADP-dependent dehydrogenase GabD)	3,2

Supplementary Table 3. List of the strains and plasmids used in this work.

Strain	Description	Reference
E. coli **042**	EAEC clinical isolate	Prof. I. Henderson
E. coli **042 Δ*lacZ***	*E. coli* 042 mutant for the *lacZ* gene	This work
E. coli **5K Rif**	*F^-, hsdR, hsdM, thr,m thi, rpsL, leu, lacZ*, Rif^r	[3]
E. coli **55989**	EAEC clinical isolate	[4]
E. coli **DH5α**	*fhuA2 lac(del)U169 phoA glnV44 Φ80' lacZ(del)M15 gyrA96 recA1 relA1 endA1 thi-1 hsdR17*	[5]
E. coli **042 pAA2-**	*E. coli* 042cured from pAA2 plasmid	[6]
E. coli **042 Δ*lacZ aggR::lacZ***	*E. coli* 042 Δ*lacZ* with transcriptional fusion of *lacZ* reporter gene under the control of *aggR* promoter	[6]
E. coli **042 Δ*lacZ aggR3'UTR::lacZ***	*E. coli* 042 Δ*lacZ* with transcriptional fusion of *lacZ* reporter gene located after the end of the coding region of the *aggR* gene	This work
E. coli **042 *aggR-FLAG***	*E. coli* 042 with the epitope FLAG inserted in the C-terminal of the AggR protein	[6]
E. coli **042 *aafA-FLAG***	*E. coli* 042 with the epitope FLAG inserted in the C-terminal of the AafA protein	[6]
E. coli **042 *aap-FLAG***	*E. coli* 042 with the epitope FLAG inserted in the C-terminal of the Aap protein	This work
E. coli **042 *aggR::Km***	*E. coli* 042 with a kanamycin resistance cassette inserted in the coding region of the *aggR* gene	This work

E. coli* 042 Δ*aggR	*E. coli* 042 mutant for the *aggR* gene	6
E. coli* 042*aggR+Km-r3'UTR	*E. coli* 042 with a kanamycin resistance cassette inserted after the stop codon of the *aggR* open reading frame	This work
E. coli* 042 *aggR+FRT3'UTR	*E. coli* 042 with a FRT scar inserted after the stop codon of the *aggR* gene	This work
E. coli* 042 *aggR+FRT3'UTR aar	*E. coli* 042 with a FRT scar inserted after the stop codon of the *aggR* gene and mutant for the *aar* gene	This work
E. coli* 042 *aggR+FRT3'UTR ast fad	*E. coli* 042 with a FRT scar inserted after the stop codon of the *aggR* gene and mutant for the *ast* and *fad* operons	This work
E. coli* 042 Δ*3'UTRaggR	*E. coli* 042 mutant for the 3'UTR region of the *aggR* gene	This work
E. coli* 042 Δ*IRL* IS*1A	*E. coli* 042 mutant for the IRL sequence of the IS*1A* located downstream the *aggR* gene	This work
E. coli* 042 Δ*3'UTRaggR* Δ*IRL* IS*1A	*E. coli* 042 mutant for the 3'UTR region of the *aggR* gene and the IRL sequence of the IS*1A* located downstream the *aggR* gene	This work
E. coli* 042 ΔIS*1A	*E. coli* 042 mutant for the insertion element IS*1A* located downstream the *aggR* gene	This work
E. coli* 042 Δ*RNase E	*E. coli* 042 mutant for the C-terminal region of the RNase E protein	This work
E. coli* 042 Δ*Pnp	*E. coli* 042 mutant for the polynucleotide phosphorylase protein	This work
E. coli* 55989 *aggR + Km-r 3'UTR	*E. coli* 55989 with a kanamycin resistance cassette inserted after the stop codon of the *aggR* open reading frame	This work
E. coli* 55989 *aggR+FRT3'UTR	*E. coli* 55989 with a FRT scar inserted after the stop codon of the *aggR* gene	This work

35

**Plasmid**	**Description**	**Reference**
**pKD3**	*bla* (Cb^r) FRT *cat* FRT PS1 PS2 oriR6K Cm^r	7
**pKD4**	*bla*(Cb^r) FRT *ahp* FRT PS1 PS2 oriR6K Km^r	7
**pKD46**	*bla* (Cb^r) P_{BAD}*gam bet exo* pSC101 oriTS	7
**pCP20**	*bla*(Cb^r) *cat* λ *cI*857 *l*P_R*flp* pSC101 oriTS	8
**pSUB11**	R6KoriV 3XFLAG-Km^r	9
**pKG136**	*ahp FRT lacZY+ t*$_{his}$oriR6K	10

Supplementary Table 4. List of the oligonucleotides used in this work.

Oligonucleotie	Sequence (5'-3')
**aggR 042 p1**	5'ACATTTTTTTCATGTGAGAATGATATGAAATTAAAACAAAACG TGTAGGCTGGAGCTGCTTC 3'
**aggR 042 p2**	5'TTATTGGCTTTTAAAATAAGTCAAGAATTGTTTTGGTGTTATCA TATGAATATCCTCCTTAGT 3'
**aggR 042 p1 up**	5' GCTGCAATTAAGATACAACCCC 3'
**aggR 042 3UTR p1**	5'ATAACACCAAAACAATTCTTGACTTATTTTAAAAGCCAATAAG TGTAGGCTGGAGCTGCTTC 3'
**aggR 042 3UTR p2**	5'ATATGTTTATAGCAATCTCAAATAATGATATGAAACATGTTTCA TATGAATATCCTCCTTAGT 3'
**aggR 042 3UTR p1 up**	5' CGCAGATTGCCTGATAAAGAC 3'
**aggR 042 3UTR p2 down**	5' TTGCCGTTACGCACCACTCCG 3'
**aap FLAG p1**	5'AGTCCAAAAATATCGTGCTCTAACCGAATGGGTTAAAGACTAC AAAGACCATGACGG 3'
**aap FLAG p2**	5'GTGGAGAGTTGAAATTTTAGCTAGAGCTAGATATTATTTAACC GACTACAAAGACCATGACGG 3'
**aap FLAG p1 up**	5' GCTAGCCTTCTAAAAGGAGGG 3'
**aap FLAG p2 down**	5' ATGCGGACAACAGTGTTTACG 3'
**aggR 55989 3UTR p1**	5'ATAACACCAAAACAATTCTTGACTTATTTTAAAAGCCAATGAG TGTAGGCTGGAGCTGCTTC 3'
**aggR 55989 3UTR p2**	5'ATATGTTTATAGCAATCTCAAATAATGATATAAAACATATTTCA TATGAATATCCTCCTTAGT 3'
**aggR 55989 3UTR p1 up**	5' GACTGTTGCGATCGTGAAGCT 3'
**aggR 55989 3UTR p2 down**	5' ACGGTGGCAGTCACGGTAGCG 3'
**aggR IRL p1**	5'TCATATCATTATTTGAGATTGCTATAAACATATTGAGATGGCG TGTAGGCTGGAGCTGCTTC 3'
**aggR IRL p2**	5'AGTAGCTGAACAGGAGGGACAGCTGATAGAAACAGAAGCCA CCATATGAATATCCTCCTTAGT 3'
**aggR IS1a p1**	5'ATTATTTGAGATTGCTATAAACATATTGAGATGGCTGAAGTTG TGTAGGCTGGAGCTGCTTC 3'
**aggR IS1a p2**	5'GTTTAGCGACTCGATGGAATTCGTTGTATAGATCACTTTGCGC ATATGAATATCCTCCTTAGT 3'
**aggR IS1a p2 down**	5' CTCAATTACACCGGCTATGCC 3'
**aar p1**	5'TCTTATCATGTTATAAATTCCAGAAAAGAGAACATTGTATTGG TGTAGGCTGGAGCTGCTTC 3'

**aar p2**	5'TTAGGCCAGTCTAGACAGTTTTTGTTGACGACTACACTTTCATATGAATATCCTCCTTAGT 3'
**aar p1 up**	5' GCCCTTTCCCATAATCTCTCA 3'
**aar p2 down**	5' GATCGCACAATCCGGGCTGAG 3'
**ast p1**	5'CCCTGGGCTGGATTTTTCCGATGAGGTGGTGCGATGAACGCCGTGTAGGCTGGAGCTGCTTC 3'
**ast p2**	5'TAATACCCGCAGAATGATTTCTGCGGGTAAGTATTAGCTTATCATATGAATATCCTCCTTAGT 3'
**ast p1up**	5' CGCTTACCGGCGCTGCCAGTA 3'
**ast p2 down**	5' TCAATTAATCAGAGCAACGGT 3'
**fad p1**	5'GACCAGATCACCTTGCGGATTCAGGAGACTGACATGCTTTACGTGTAGGCTGGAGCTGCTTC 3'
**fad p2**	5'CGGATAAGGCGTCACGCCGCATCCGGCAAGTGGTTAAATCCGCATATGAATATCCTCCTTAGT 3'
**fad p1 up**	5' CTGCCGAGCGTGATCAGATCG 3'
**fad p2 down**	5' GTCGCATCCGGCAATCGGTGC 3'
**RNase E C/Terminal p1**	5'GAAACCCCGCACTACCATGTGCTGCGCGTGCGCAAAGGGGAAGTGTAGGCTGGAGCTGCTTC 3'
**RNase E C/Terminal p2**	5'AAGCCCTGGCAGTTACCAGGGCTTGATTGCTTGAGCTAATTACATATGAATATCCTCCTTAGT 3'
**RNase E C/Terminal p1 up**	5' GTCAGCGCCTGAGCCCATCAT 3'
**RNase E C/Terminal p2 down**	5' CTTCAGCTGCGGCTCTGGCGG 3'
**Pnp p1**	5'TACCCACATTGGGCTGGGTTAGGGTTGTCATTAGTCGCGAGGGTGTAGGCTGGAGCTGCTTC 3'
**Pnp p2**	5'CGGTTAAAAGCCCCCCGCCGCAGCGGAGGGCAAATGGCAACCCATATGAATATCCTCCTTAGT 3'
**Pnp p1 up**	5' GCGCCTGGGTCTGCGTCGCTA 3'
**Pnp p2 down**	5' TGCCAGAATCACTTCCTGCTG 3'
**aggR 3'**	5' GAATTGTTTTGGTGTTATGCCA 3'
**aggR 3' 1.2**	5' GGCTTTTAAAATAAGTCAAGA 3'
**aggR antisense**	5' CCTTATGCAATCAAGAATGAG 3'
**5S**	5' CTACGGCGTTTCACTTCTGAGTTC 3'
**aggR walking-PCR c1**	5' GTCCGAATTGGTCAAAAGGAA 3'
**aggR walking-PCR c2**	5' AAGCCTAATGAAATATGATGT 3'
**aggR walking-PCR 1**	5' CCTTATGCAATCAAGAATGAG 3'
**aggR walking-PCR 2**	5' AAGCCTAATGAAATATGATGT 3'
**aggR walking-PCR 3**	5' CTAAATCAGTAAGTTGGCAGC 3'
**aggR walking-PCR 4**	5' GTGACGTAAAATCGTGTTGAG 3'
**aggR walking-PCR 5**	5' CGACAGCGACTTCCGTCCCAG 3'

**aggR walking-PCR 6**	5’ CTGCGCTGATGCTGGTATGCG 3’
**gapA042 RT Fw**	5’ TTTCCGTGCTGCTCAGAAAC 3’
**gapA042 RT Rv**	5’ GTCAACACCAACTTCGTCCC 3’
Il1bFW	5´TGTGAAATGCCACCTTTTGA3´
Il1bRV	5´GGTCAAAGGTTTGGAAGCAG3´
Il6FW	5´ACCAGAGGAAATTTTCAATAGGC3´
Il6RV	5´TGATGCACTTGCAGAAAACA3´
Hprt1FW	5´TGGATACAGGCCAGACTTTGTT3´
Hprt1RV	5´CAGATTCAACTTGCGCTCATC3´

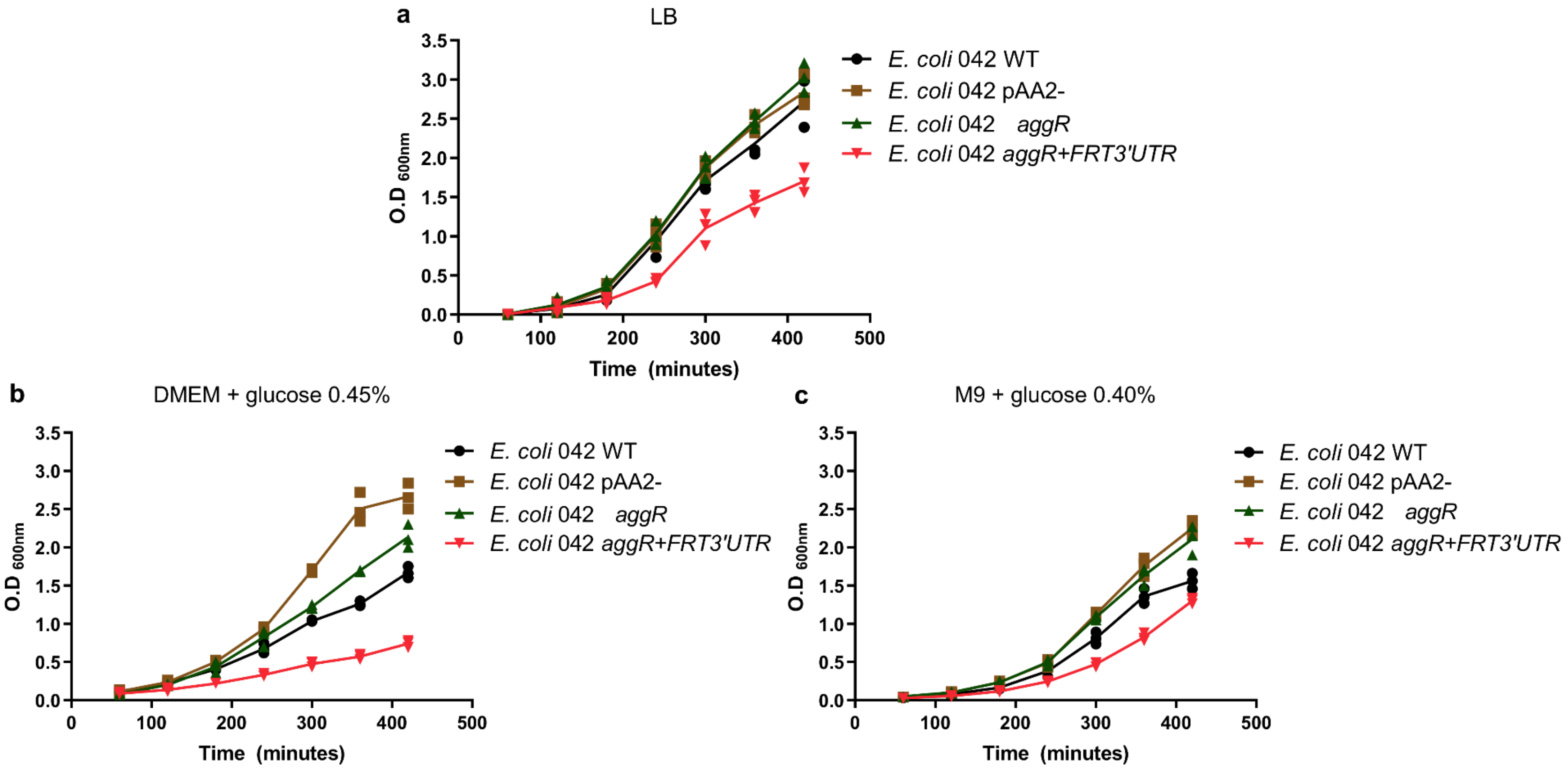

Supplementary Figure 1. A FRT sequence DNA insertion in the 3'UTR of the *aggR* gene results in a reduced growth rate. Growth curves of *E. coli* strains 042 wt, pAA2-, Δ*aggR* and *aggR+FRT3'UTR* in (a) LB medium, (b) DMEM + glucose 0.45% and (c) M9 minimal medium + glucose 0.4% at 37°C. Error bars correspond to the standard deviation of three independent biological replicates.

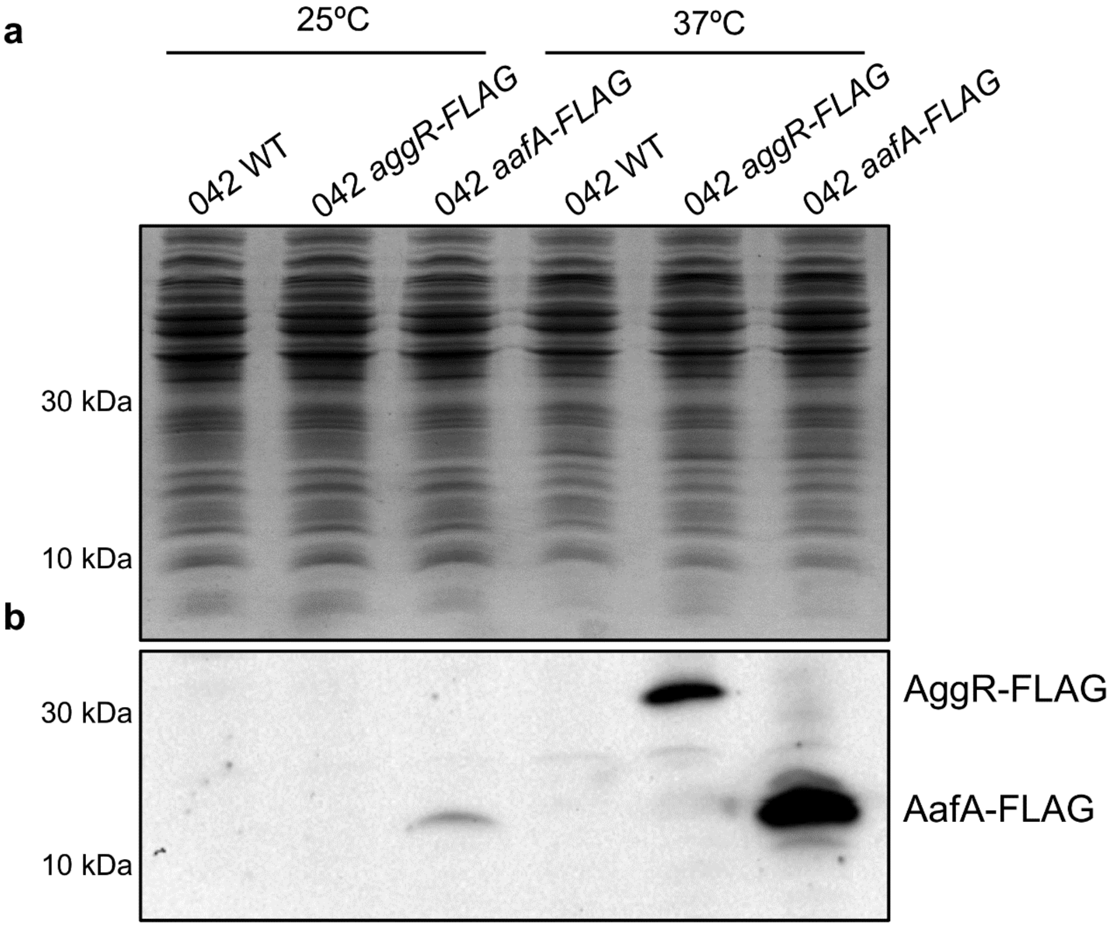

Supplementary Figure 2. AggR and AafA protein expression is temperature regulated. (a) Corresponds to the total cell extract loaded per slot and stained with Coomassie blue (loading control). (b) Western blot analysis of the 042 *aggR*-Flag and *aafA*-Flag strains grown in LB medium at 25°C and 37°C until the OD_{600} reached 2.0. Flag-tagged AggR and AafA proteins were detected using anti-Flag antibody, as indicated in the figure. The experiment was repeated three times, and a representative experiment results is presented.

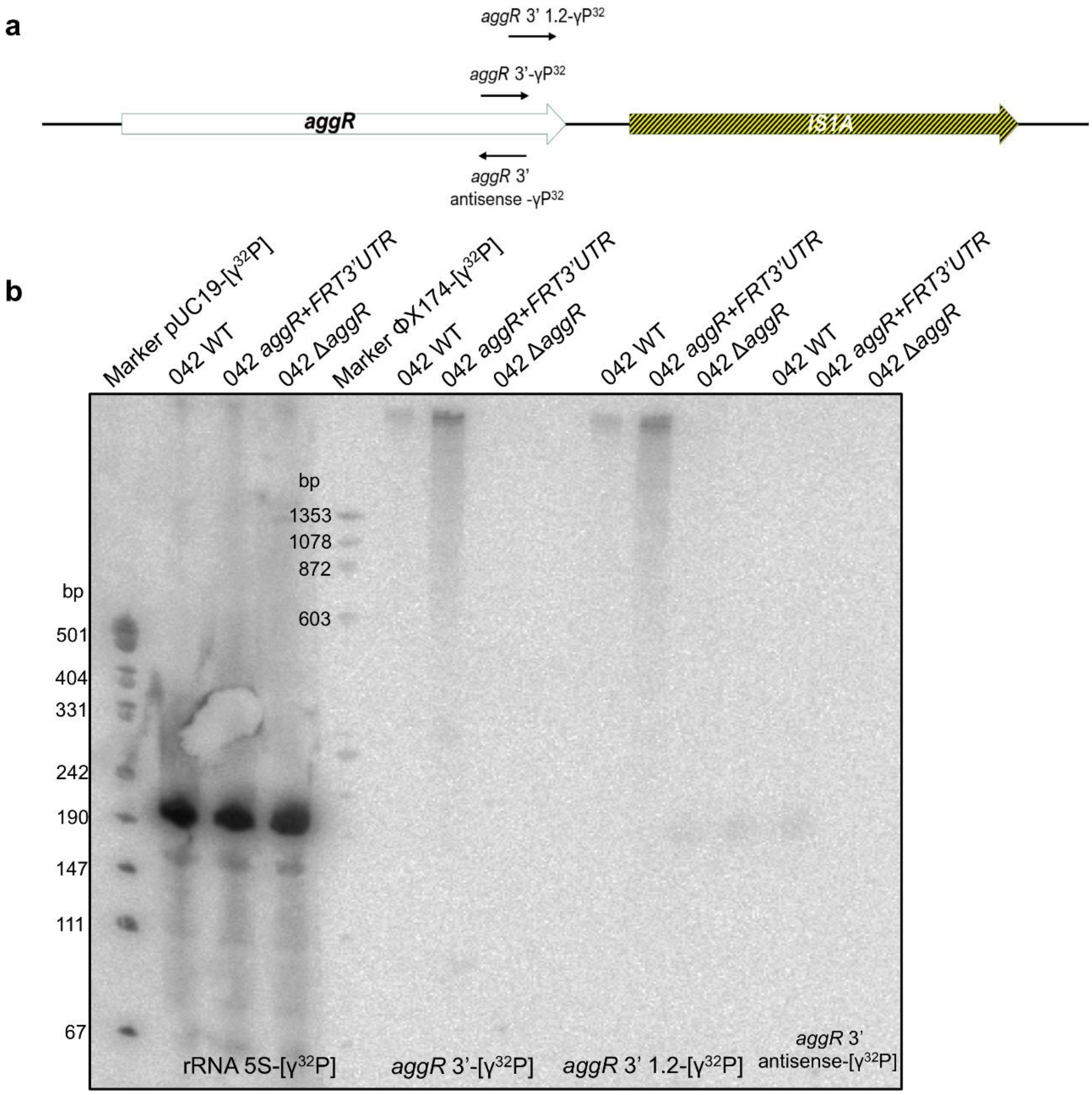

Supplementary Figure 3. Northern blot analysis of the *aggR* transcript. (a) Diagram showing the localization of the probes used for the Northern blot analysis of *aggR* mRNA. (b) Full image of a representative Northern blot of the *aggR* transcript. Molecular weight markers are shown.

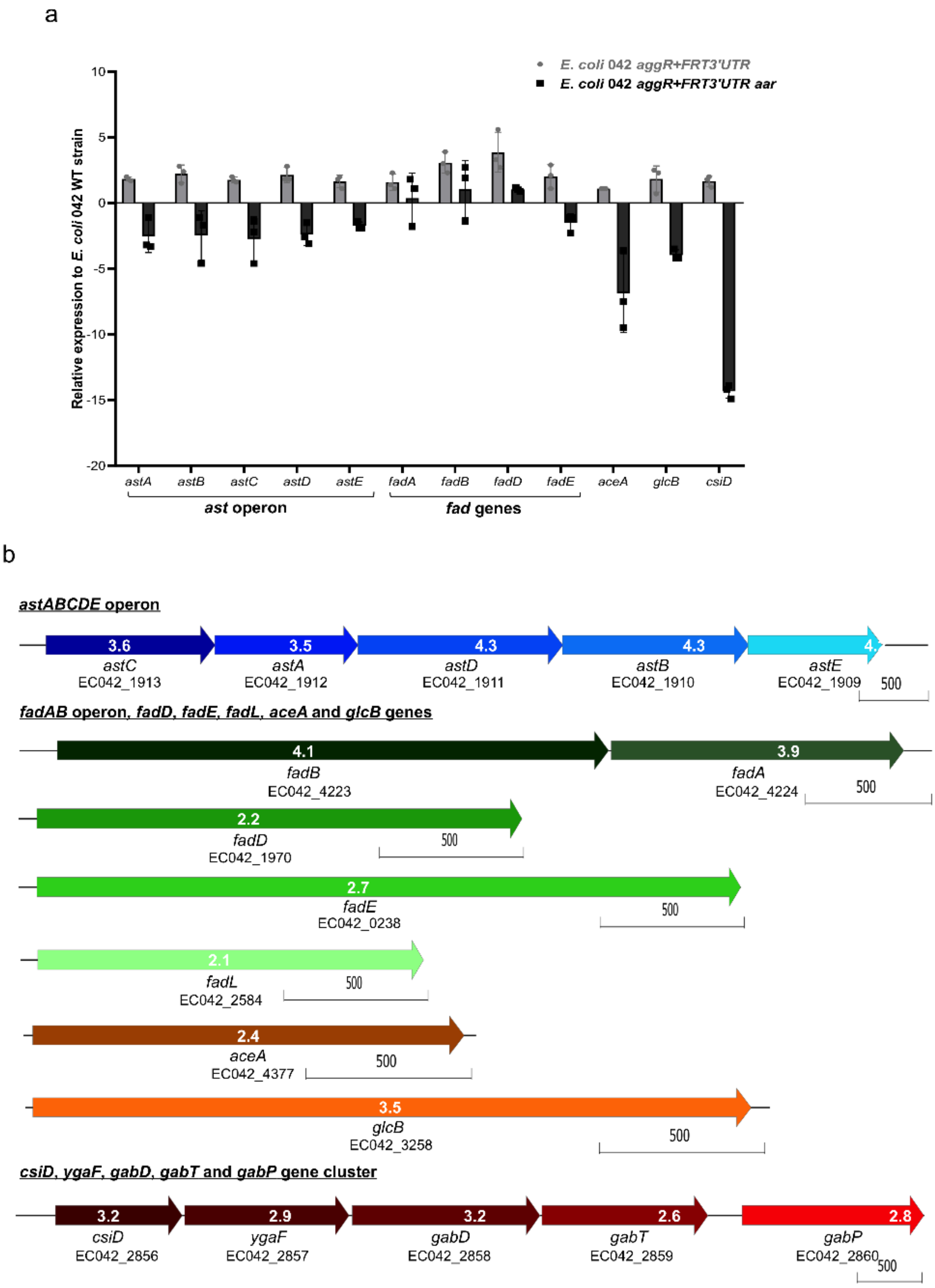

Supplementary Figure 4. Overexpression of metabolic genes in the 042 *aggR+FRT 3'UTR* and derivatives. (a) Overexpression of metabolic genes in the 042 *aggR+FRT*

3'UTR and *its aggR+FRT 3'UTR aar* derivative compared to the wt strain. Relative expression of *ast*, *fad*, *aceA*, *glcB* and *csiD* genes quantified by RT-qPCR in *E. coli* 042 *aggR+FRT3'UTR* and its *aggR+FRT3'UTR aar* derivative. The bar shows the arithmetic mean of the results of three independent experiments and the error bar indicates the standard deviation. (b) Overexpression of metabolic genes in the 042 *aggR+FRT 3'UTR* mutant compared to the wt strain. Diagram showing the metabolic pathways leading to arginine degradation (*ast*-encoded enzymes), GABA degradation (*csiD-, ygaF*-, and *gabDTP*-encoded enzymes) and fatty acid degradation (*fad*-and *ace*-encoded enzymes). Foldchange values for each gene in the 042 *aggR+FRT3'UTR* mutant compared to the wt strain are shown.

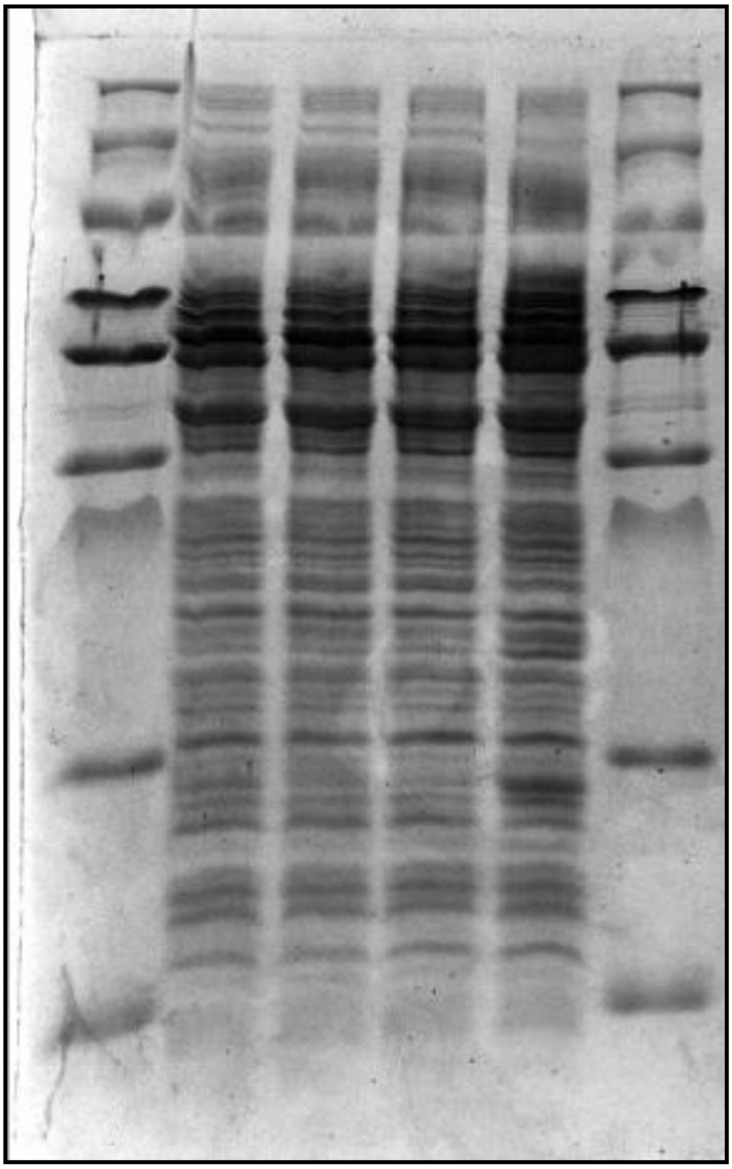

Supplementary Figure 5. Uncropped SDS-PAGE image corresponding to the Figure 2a.

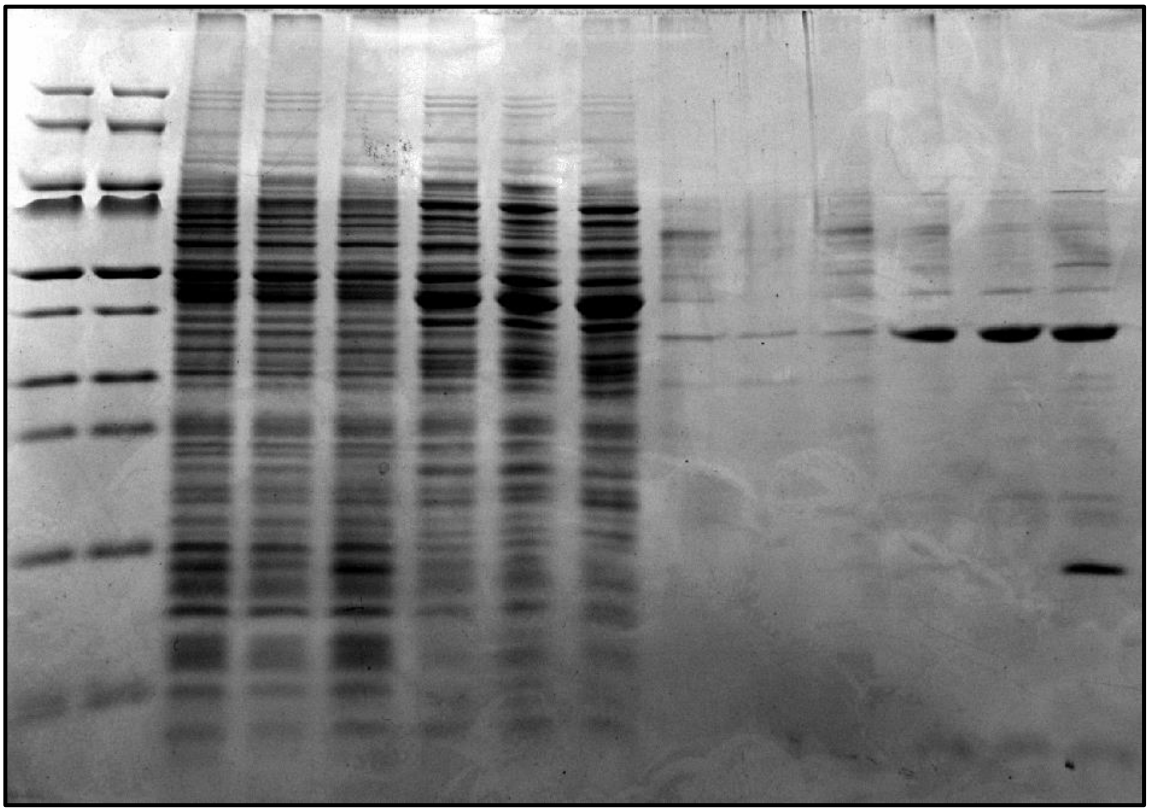

Supplementary Figure 6. Uncropped SDS-PAGE image corresponding to the Figure 2b.

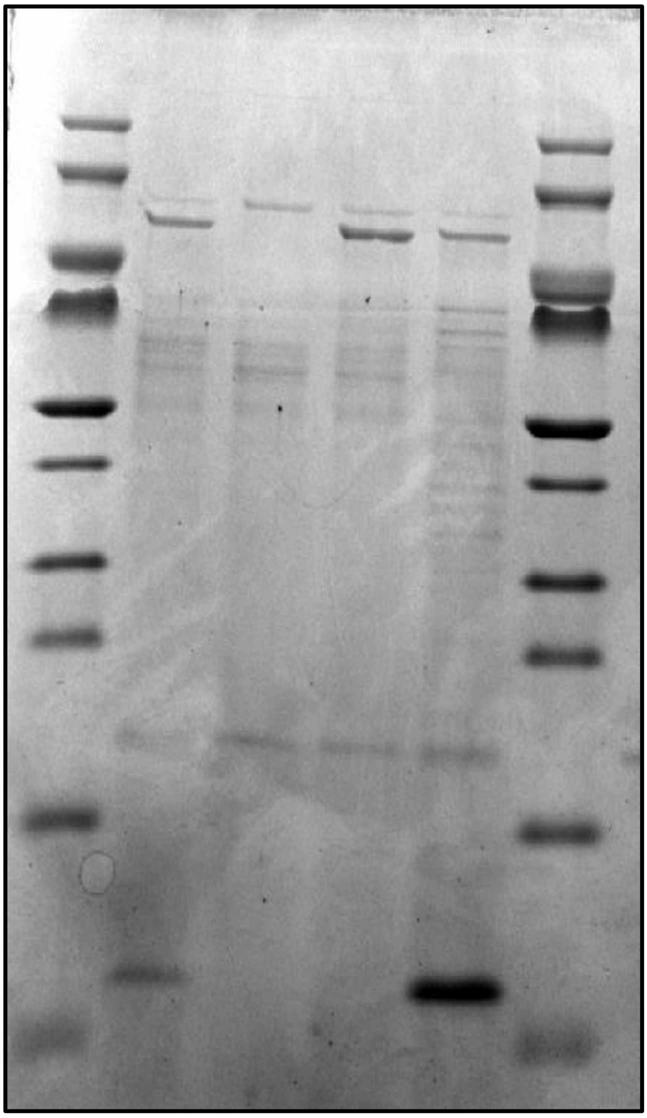

Supplementary Figure 7. Uncropped SDS-PAGE image corresponding to the Figure 2c.

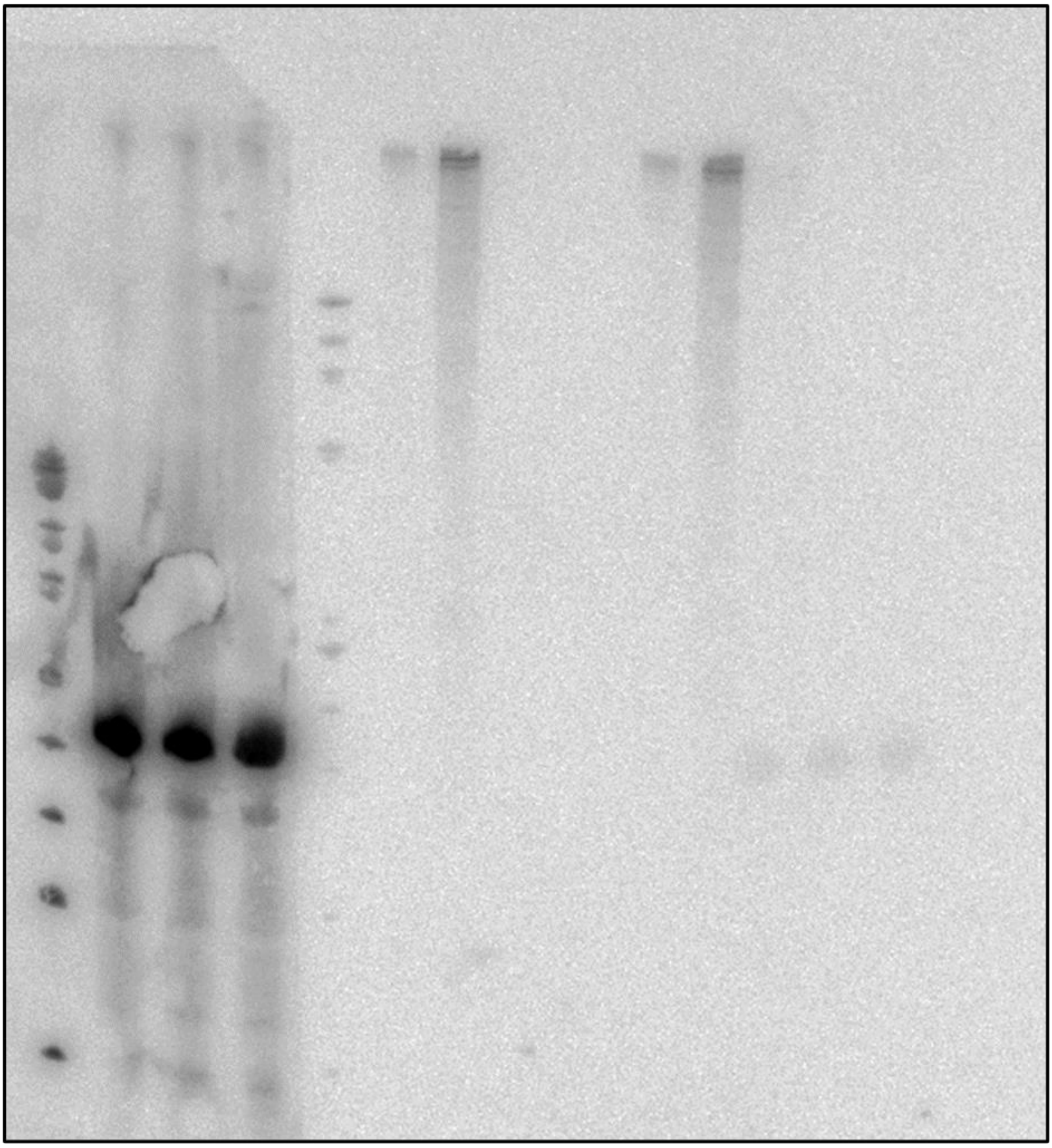

Supplementary Figure 8. Uncropped northern blot image corresponding to the Figure 3.

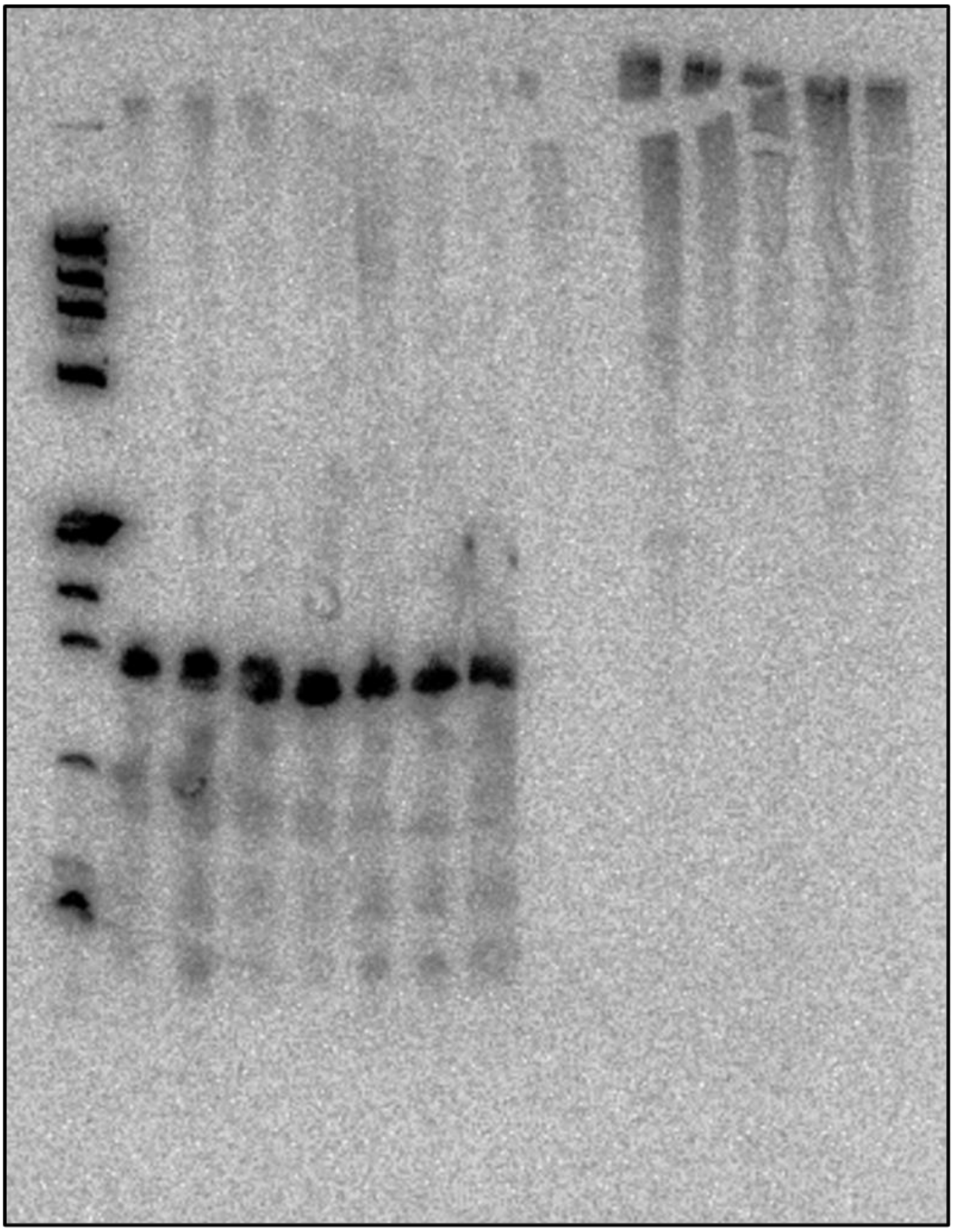

Supplementary Figure 9. Uncropped northern blot image corresponding to the Figure 6.

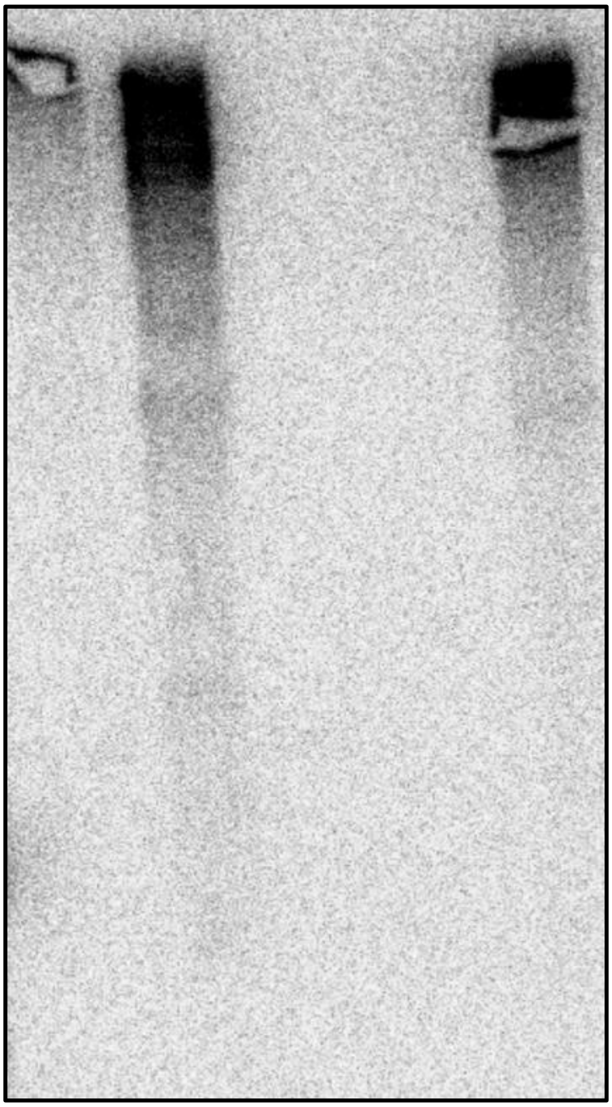

Supplementary Figure 10. Uncropped northern blot image corresponding to the Figure 8a.

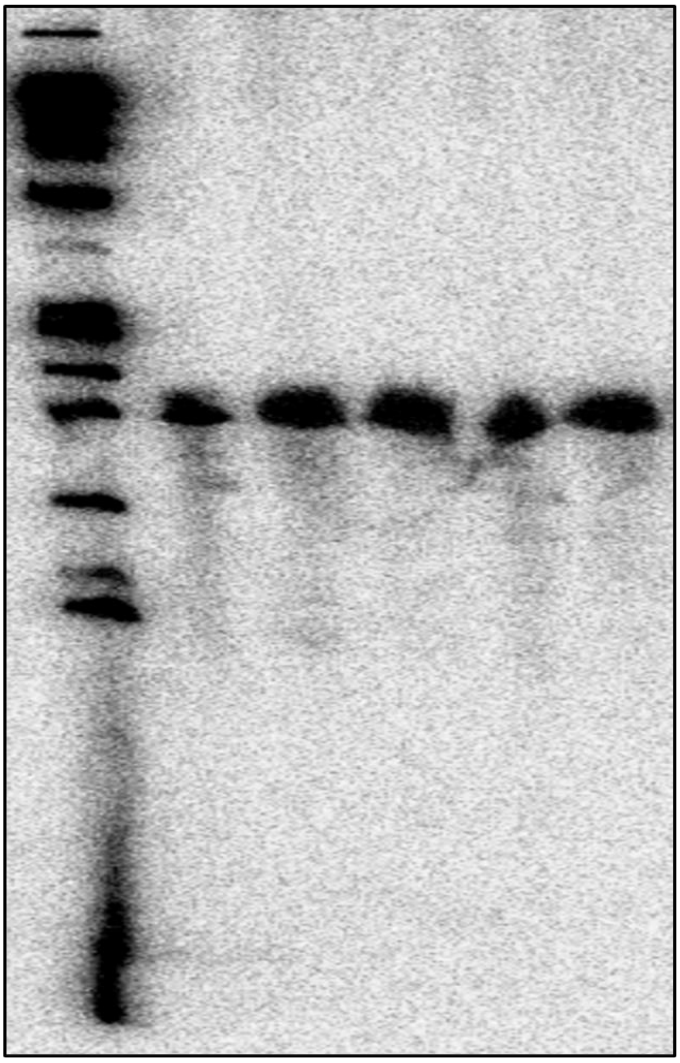

Supplementary Figure 11. Uncropped northern blot image corresponding to the Figure 8b.

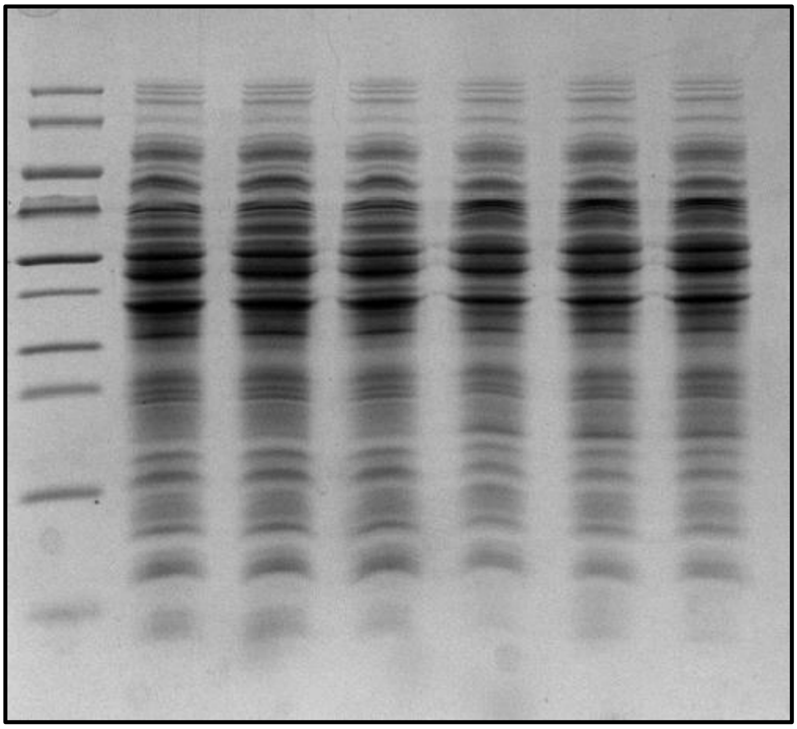

Supplementary Figure 12. Uncropped SDS-PAGE image corresponding to the Supplementary Figure 2a.

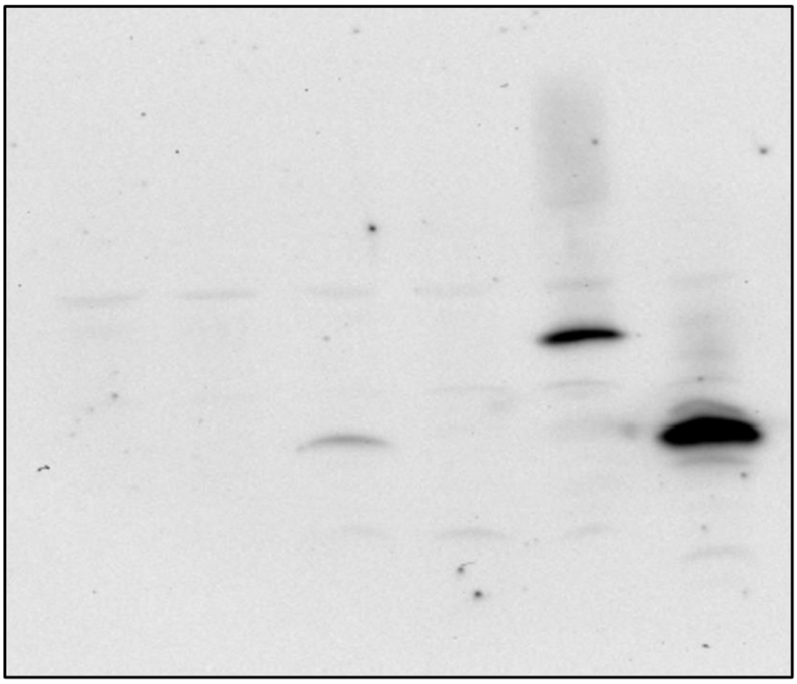

Supplementary Figure 13. Uncropped western blot image corresponding to the Supplementary Figure 2b.

Supplementary references

1. Yasir, M. *et al.* Organization and architecture of AggR-dependent promoters from enteroaggregative *Escherichia coli*. *Mol. Microbiol.* **111**, 534–551 (2019).
2. Morin, N., Santiago, A. E., Ernst, R. K., Guillot, S. J. & Nataro, J. P. Characterization of the AggR regulon in enteroaggregative *Escherichia coli*. *Infect. Immun.* **81**, 122–132 (2013).
3. Nieto, J. ., Prenafeta, A., Miquelay, E., Torrades, S. & Juarez, A. Sequence, identification and effect on conjugation of the *rmoA* gene of plasmid R100-1. *FEMS Microbiol. Lett.* **169**, 59–66 (1998).
4. Bernier, C., Gounon, P. & Le Bouguénec, C. Identification of an aggregative adhesion fimbria (AAF) type III-encoding operon in enteroaggregative *Escherichia coli* as a sensitive probe for detecting the AAF-encoding operon family. *Infect. Immun.* **70**, 4302–4311 (2002).
5. Taylor, R. G., Walker, D. C. & Mclnnes, R. R. *E.coli* host strains significantly affect the quality of small scale plasmid DNA preparations used for sequencing. *Nucleic Acids Res.* **21**, 1677–1678 (1993).
6. Hüttener, M., Prieto, A., Espelt, J., Bernabeu, M. & Juárez, A. Stringent response and AggR-dependent virulence regulation in the enteroaggregative *Escherichia coli* strain 042. *Front. Microbiol.* **9**, (2018).
7. Datsenko, K. A. & Wanner, B. L. One-step inactivation of chromosomal genes in *Escherichia coli* K-12 using PCR products. *Proc. Natl. Acad. Sci. U. S. A.* **97**, 6640–6645 (2000).
8. Cherepanov, P. P. & Wackernagel, W. Gene disruption in *Escherichia coli*: TcR and KmR cassettes with the option of Flp-catalyzed excision of the antibiotic-resistance determinant. *Gene* **158**, 9–14 (1995).
9. Uzzau, S., Figueroa-Bossi, N., Rubino, S. & Bossi, L. Epitope tagging of chromosomal genes in *Salmonella*. *Proc. Natl. Acad. Sci. U. S. A.* **98**, 15264–15269 (2001).
10. Ellermeier, C. D., Janakiraman, A. & Slauch, J. M. Construction of targeted single copy *lac* fusions using λ Red and FLP-mediated site-specific recombination in bacteria. *Gene* **290**, 153–161 (2002).
