# Supplementary material for: Modulation of AggR levels reveals features of virulence regulation in enteroaggregative E. coli
Source: Commun Biol. 2021 Nov 16;4:1295. doi: 10.1038/s42003-021-02820-9 (PMC8595720; doi:10.1038/s42003-021-02820-9)
Supplement: Supplementary file 3 — Description of Additional Supplementary Files [file 42003_2021_2820_MOESM3_ESM.pdf]

Description of Additional Supplementary Files

File name: Supplementary Data 1.
Description: Source data for the graphs in the main figures.
