# Supplementary material for: Modulation of AggR levels reveals features of virulence regulation in enteroaggregative E. coli
Source: Commun Biol. 2021 Nov 16;4:1295. doi: 10.1038/s42003-021-02820-9 (PMC8595720; doi:10.1038/s42003-021-02820-9)
Supplement: Supplementary file 5 — Reporting Summary [file 42003_2021_2820_MOESM5_ESM.pdf]

nature research

Corresponding author(s): MÁRIO HÜTTENER and ANTONIO JUÁREZ

Last updated by author(s): Oct 13, 2021

Reporting Summary

Nature Research wishes to improve the reproducibility of the work that we publish. This form provides structure for consistency and transparency in reporting. For further information on Nature Research policies, see our Editorial Policies and the Editorial Policy Checklist.

Statistics

For all statistical analyses, confirm that the following items are present in the figure legend, table legend, main text, or Methods section.

n/a	Confirmed	
☐	☒	The exact sample size (*n*) for each experimental group/condition, given as a discrete number and unit of measurement
☐	☒	A statement on whether measurements were taken from distinct samples or whether the same sample was measured repeatedly
☐	☒	The statistical test(s) used AND whether they are one- or two-sided<br>*Only common tests should be described solely by name; describe more complex techniques in the Methods section.*
☒	☐	A description of all covariates tested
☒	☐	A description of any assumptions or corrections, such as tests of normality and adjustment for multiple comparisons
☒	☐	A full description of the statistical parameters including central tendency (e.g. means) or other basic estimates (e.g. regression coefficient) AND variation (e.g. standard deviation) or associated estimates of uncertainty (e.g. confidence intervals)
☐	☒	For null hypothesis testing, the test statistic (e.g. *F*, *t*, *r*) with confidence intervals, effect sizes, degrees of freedom and *P* value noted<br>*Give P values as exact values whenever suitable.*
☒	☐	For Bayesian analysis, information on the choice of priors and Markov chain Monte Carlo settings
☒	☐	For hierarchical and complex designs, identification of the appropriate level for tests and full reporting of outcomes
☒	☐	Estimates of effect sizes (e.g. Cohen's *d*, Pearson's *r*), indicating how they were calculated

Our web collection on statistics for biologists contains articles on many of the points above.

Software and code

Policy information about availability of computer code

Data collection	qRT-PCR data was collected with ABI Prism 7700 sequence detection system (Applied Biosystems). Western blot images were collected with Molecular Imager ChemiDoc XRS system and Quantity One software (Bio-Rad). Northern blot images were collected with a phosphoimager Molecular imager FX pro plus multiImager system (Bio-Rad).
Data analysis	RhoTermPredict software for analyzing the presence of Rho-dependent transcriptional terminators and the Arnold software for analyzing for the presence of Rho-independent transcription terminators. Statistical analysis was performed using GraphPadPrism version 8.0. qRT-PCR data was analysed with StepOne Software version 2.3.

For manuscripts utilizing custom algorithms or software that are central to the research but not yet described in published literature, software must be made available to editors and reviewers. We strongly encourage code deposition in a community repository (e.g. GitHub). See the Nature Research guidelines for submitting code & software for further information.

Data

Policy information about availability of data

All manuscripts must include a data availability statement. This statement should provide the following information, where applicable:

- Accession codes, unique identifiers, or web links for publicly available datasets
- A list of figures that have associated raw data
- A description of any restrictions on data availability

The RNA-sequencing reads have been deposited in the Gene Expression Omnibus (GEO) Sequence Read Archive of the National Center for Biotechnology Information (GSE160448) under accession numbers GSM4873501 and GSM4873502.

Field-specific reporting

Please select the one below that is the best fit for your research. If you are not sure, read the appropriate sections before making your selection.

- [x] Life sciences
- [ ] Behavioural & social sciences
- [ ] Ecological, evolutionary & environmental sciences

For a reference copy of the document with all sections, see nature.com/documents/nr-reporting-summary-flat.pdf

Life sciences study design

All studies must disclose on these points even when the disclosure is negative.

Sample size	All in vitro experiments and their statistical analyses were performed using at least 3 biological replicates. For in vivo experiments, the samples from animals (C57BL/6 mice) were collected from 10 mice from each group.
Data exclusions	There were no excluded samples or data from the analysis in this study.
Replication	Each experiment was repeated at least three time as indicated in the figure legend. All experimental data was reproducible with similar observation in each attempt.
Randomization	We randomly choose mice for each experiment group and also randomly chose the control mice with same sex and similar date birth.
Blinding	N/A

Reporting for specific materials, systems and methods

We require information from authors about some types of materials, experimental systems and methods used in many studies. Here, indicate whether each material, system or method listed is relevant to your study. If you are not sure if a list item applies to your research, read the appropriate section before selecting a response.

Materials & experimental systems

n/a	Involved in the study	
[ ]	[x]	Antibodies
[ ]	[x]	Eukaryotic cell lines
[x]	[ ]	Palaeontology and archaeology
[ ]	[x]	Animals and other organisms
[x]	[ ]	Human research participants
[x]	[ ]	Clinical data
[x]	[ ]	Dual use research of concern

Methods

n/a	Involved in the study	
[x]	[ ]	ChIP-seq
[x]	[ ]	Flow cytometry
[x]	[ ]	MRI-based neuroimaging

Antibodies

Antibodies used	Anti-Flag (Sigma-Aldrich - Cat. Nº F3165), Anti-Mouse HRP Conjugate (Promega - Cat Nº W4021).
Validation	All antibodies used in this study were obtained from commercial sources and validated according to manufacturers's instruction.

Eukaryotic cell lines

Policy information about cell lines

Cell line source(s)	Human TLR4-expressing HEK 293 cells (InvivoGen - Cat Nº hkb-htlr4).
Authentication	The cell line were purchased from InvivoGen and authenticated by the vendor.
Mycoplasma contamination	The cell line were tested Mycoplasma negative.
Commonly misidentified lines (See ICLAC register)	No misidentified cell lines were used in this study.

Animals and other organisms

Policy information about studies involving animals; ARRIVE guidelines recommended for reporting animal research

Laboratory animals	Male C57BL/6 mice (8 weeks age) Envigo (Bresso, Italy).

April 2020

Wild animals	The study did not involve wild animals.
Field-collected samples	The study did not involve samples collected from the field.
Ethics oversight	Ethics Committee for Animal Experimentation of the University of Barcelona and the Catalan government (Refs. 290/19 and 10969, respectively).

Note that full information on the approval of the study protocol must also be provided in the manuscript.
